# Associations between Breastfeeding Duration and Obesity Phenotypes and the Offsetting Effect of a Healthy Lifestyle

**DOI:** 10.3390/nu14101999

**Published:** 2022-05-10

**Authors:** Jiajia Dang, Ting Chen, Ning Ma, Yunfei Liu, Panliang Zhong, Di Shi, Yanhui Dong, Zhiyong Zou, Yinghua Ma, Yi Song, Jun Ma

**Affiliations:** 1 Institute of Child and Adolescent Health, School of Public Health, Peking University, Beijing 100191, China; dangjj@bjmu.edu.cn (J.D.); chentbjmu@163.com (T.C.); mnmaning@bjmu.edu.cn (N.M.); liuyunfei_pku@163.com (Y.L.); 1610306219@pku.edu.cn (P.Z.); 2111210123@bjmu.edu.cn (D.S.); dongyanhui@bjmu.edu.cn (Y.D.); harveyzou2002@bjmu.edu.cn (Z.Z.); yinghuama@bjmu.edu.cn (Y.M.); 2 National Health Commission Key Laboratory of Reproductive Health, Peking University, Beijing 100191, China

**Keywords:** breastfeeding, obesity phenotypes, healthy lifestyle, children and adolescents

## Abstract

*Background*: Additional metabolic indicators ought to be combined as outcome variables when exploring the impact of breastfeeding on obesity risk. Given the role of a healthy lifestyle in reducing obesity, we aimed to assess the effect of breastfeeding duration on different obesity phenotypes according to metabolic status in children and adolescents, and to explore the offsetting effect of healthy lifestyle factors on the associations between breastfeeding duration and obesity phenotypes. *Methods*: A total of 8208 eligible children and adolescents aged 7–18 years were recruited from a Chinese national cross-sectional study conducted in 2013. Anthropometric indicators were measured in the survey sites, metabolic indicators were tested from fasting blood samples, and breastfeeding duration and sociodemographic factors were collected by questionnaires. According to anthropometric and metabolic indicators, obesity phenotypes were divided into metabolic healthy normal weight (MHNW), metabolic unhealthy normal weight (MUNW), metabolic healthy obesity (MHO), and metabolic unhealthy obesity (MUO). Four common obesity risk factors (dietary consumption, physical activity, screen time, and sleep duration) were used to construct a healthy lifestyle score. Scores on the lifestyle index ranged from 0 to 4 and were further divided into unfavorable lifestyles (zero or one healthy lifestyle factor), intermediate lifestyles (two healthy lifestyle factors), and favorable lifestyle (three or four healthy lifestyle factors). Multinomial logistic regression was used to estimate the odds ratio (OR) and 95% confidence interval (95% CI) for the associations between breastfeeding duration and obesity phenotypes. Furthermore, the interaction terms of breastfeeding duration and each healthy lifestyle category were tested to explore the offsetting effect of lifestyle factors. *Results*: The prevalence of obesity among Chinese children and adolescents aged 7–18 years was 11.0%. Among the children and adolescents with obesity, the prevalence of MHO and MUO was 41.0% and 59.0%, respectively. Compared to the children and adolescents who were breastfed for 6–11 months, prolonged breastfeeding (≥12 months) increased the risks of MUNW (OR = 1.35, 95% CI: 1.19–1.52), MHO (OR = 1.61, 95% CI: 1.27–2.05), and MUO (OR = 1.46, 95% CI: 1.20–1.76). When stratified by healthy lifestyle category, there was a typical dose–response relationship between duration of breastfeeding over 12 months and MUNW, MHO, and MUO, with an increased risk of a favorable lifestyle moved to an unfavorable lifestyle. *Conclusions*: Prolonged breastfeeding (≥12 months) may be associated with increased risks of MUNW, MHO, and MUO, and the benefits of breastfeeding among children and adolescents may begin to wane around the age of 12 months. The increased risks may be largely offset by a favorable lifestyle.

## 1. Introduction

Recently, childhood obesity has been among the looming global public health issues, which could cause metabolic abnormalities, type 2 diabetes, cardiovascular diseases, and tumors in adulthood, thus representing a serious medical burden [1,2,3]. China has the largest number of children and adolescents with obesity in the world [4], where the prevalence of overweight and obesity among children and adolescents aged 6–17 years has exceeded 19% [5]. Body mass index (BMI) is a combination of weight and height, thus representing a typical way to identify obesity. However, it has been shown that individuals with the same BMI may have different status of metabolic components, including blood glucose, blood pressure, and blood lipid levels [6]. Therefore, the definition of obesity by BMI alone does not accurately reflect obesity-related metabolic status and susceptibility to metabolic diseases. There are two obesity phenotypes based on BMI combined with metabolic components. People with obesity and normal metabolic characteristics are considered as metabolic healthy obesity (MHO), while people with obesity and metabolically unhealthy status are defined as metabolic unhealthy obesity (MUO) [7,8]. MUO is associated with a higher risk of cardiovascular disease and mortality compared to MHO [9]. Given that different obesity phenotypes have varying disease risks, identifying the modifiable risks and protective factors of obesity phenotypes may contribute to future precise stratified management of obesity intervention.

Among modifiable risk factors for childhood obesity in the first 1000 days of life, breastfeeding was considered as an effective protective factor to reduce the risk of childhood obesity due to the bioactive compounds of breast milk [10,11,12,13,14,15]. However, breastfeeding as a measure to prevent overweight and obesity in children and adolescents has produced conflicting results. Some systematic reviews, most of which were conducted in European countries [16,17,18], as well as several studies from the United States [19,20,21] and Brazil [22], reported that breastfeeding duration was inversely related to the risk of obesity in children and adolescents. However, a large randomized controlled trial that promoted longer breastfeeding duration in Belarus did not show significant or meaningful changes in obesity, blood pressure, or cardiometabolic risk factors in children and adolescents [23,24,25]. Another cohort study in Japan had similar findings [26]. In addition, cohort studies in Finland and Sweden found a positive association between breastfeeding duration and obesity [27,28]. According to these findings, the association between breastfeeding duration and childhood obesity may vary by study design, ethnicities, confounding factors, and the definition of obesity itself, thus necessitating further exploration [29,30].

Previous studies that measured only BMI in children may have underestimated the true impact of breastfeeding on obesity risk [31], and more metabolic indicators need to be combined as outcome variables such as MUO with higher health damage to assess the effect of breastfeeding. More studies urgently ought to explore the associations between breastfeeding duration and obesity phenotypes. Studies have found that breastfeeding was associated with healthier eating behaviors in later childhood, such as higher consumption of vegetables and fruits and lower consumption of sugary drinks and ultra-processed foods [32,33,34]. Additionally, a large number of studies showed that a favorable lifestyle such as healthy diet, adequate sleeping duration, and physical activity established in later life would significantly decrease the risk of childhood obesity [35,36,37]. However, it remains unclear whether adherence to a healthy lifestyle could influence the associations between breastfeeding duration and obesity or obesity phenotypes.

We hypothesized that breastfeeding duration was related to obesity phenotypes, and that these associations might be affected by a healthy lifestyle. In this study, we aimed to assess the effect of breastfeeding duration on different obesity phenotypes and to investigate whether a healthy lifestyle would modify these associations among children and adolescents aged 7–18 years using the data from a Chinese national cross-sectional study in 2013.

## 2. Materials and Methods

### 2.1. Study Design and Population

**Figure 1:**
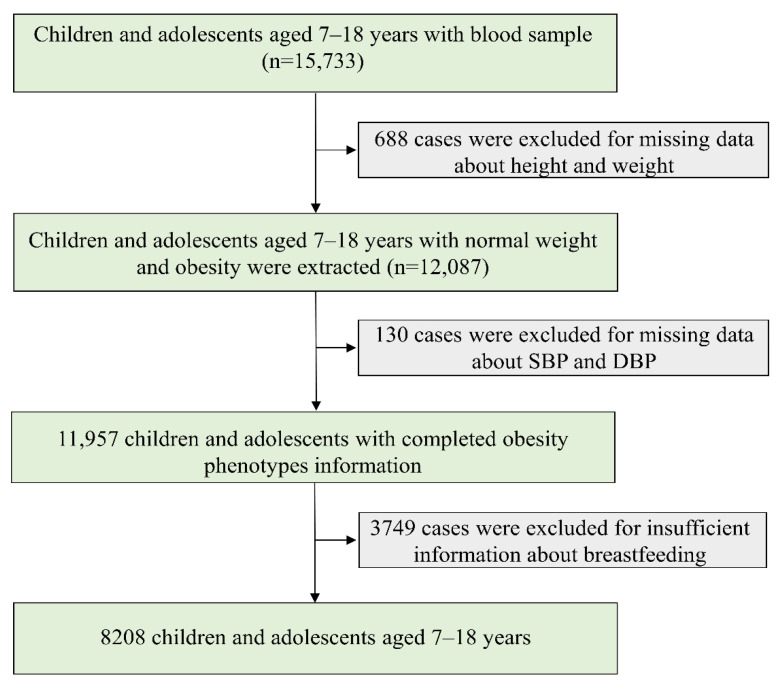
Flow chart of data. SBP, systolic blood pressure; DBP, diastolic blood pressure.

Data were collected and maintained by a national cross-sectional study conducted in September 2013. Children and adolescents aged 7–18 years from seven provinces, namely, Tianjin, Shanghai, Chongqing, Liaoning, Hunan, Ningxia, and Guangdong, were selected by a multistage cluster random sampling method. The seven provinces in this study came from different economic levels and different geographical regions of China. The detailed description of the study design was reported in a previous study [38]. In brief, 3–4 districts were randomly selected from each province, with 12–16 schools randomly selected from each district, and 2–3 classes per grade randomly selected from each school. A total of 15,733 children and adolescents aged 7–18 years with a blood sample were included (Figure 1). In our study, children and adolescents with normal weight and obesity were extracted, and cases with missing data about systolic blood pressure (SBP), diastolic blood pressure (DBP), and breastfeeding were excluded. The remaining 8208 children and adolescents were included in final analysis.

### 2.2. Data Collection and Questionnaire Survey

#### 2.2.1. Anthropometric Measurements

All anthropometric measurements were conducted using standardized instruments and procedures. Height (cm) was measured to the nearest 0.1 cm by a portable stadiometer (model TZG, China) where participants need to stand straight with light clothes and without shoes. Body weight (kg) was assessed by a lever-type weight scale (model RGT-140, China) to the nearest 0.1 kg. Body mass index (BMI) was then calculated as weight (kg) divided by the square of the height (m). Children and adolescents were required to sit quietly for at least 5 min prior to the first measurement of SBP (mmHg) and DBP (mmHg) using a mercury sphygmomanometer (model XJ1ID, Shanghai Medical Instruments Co., Ltd., Shanghai, China). Each indicator was measured twice, and the mean of the two measurements was calculated for the final analysis. Specifically, a 5 min break was allowed between the two measurements of blood pressure.

#### 2.2.2. Blood Sample Collection and Detection

Venous blood was collected by venipuncture after fasting for 12 h. After centrifuging at 3000 rpm for 10 min, serum was collected and transported to the experimental center at low temperature (−80 °C). Blood biochemical analyses were performed by a biomedical analysis company certified by Peking University [39]. Fasting plasma glucose (FPG), triglyceride (TG), and high-density lipoprotein cholesterol (HDL-C) were analyzed using the hexokinase method, enzymatic method, and clearance method, respectively.

#### 2.2.3. Questionnaire Survey

A self-administered questionnaire of children and adolescents was used to obtain information on basic characteristics (e.g., age, sex, residence, and single-child status), dietary consumption (meat, sugar-sweetened beverage, fruit, and vegetable consumption), physical activity, screen time, and sleep duration. The questionnaires were revised in the early stage of our project and validated by experts, before being deemed feasible and acceptable for children and parents. Some questionnaires (3%) from the same participants were re-examined within a week. All questionnaires were also checked for logicality and integrity. Children aged 7–9 years were administered the questionnaire with the assistance of their parents. We gathered the frequency (day) and amount (servings) of each food over the course of a week. The average daily intake of a single food was calculated using the following equation: (day × quantity in each of those days)/7 [40].

A self-administered parent questionnaire was used to obtain information on breastfeeding duration, birth weight, delivery time, and delivery model for children and adolescents, education level and tobacco and alcohol consumption for parents, maternal age at delivery, and family household income. Family history of diseases was considered as having a family history of obesity, hypertension, diabetes mellitus, or cerebrovascular disease when either paternal or maternal diagnosis was self-reported.

### 2.3. Definition and Categorization of Indicators

#### 2.3.1. Obesity Phenotypes

Obesity phenotypes included metabolic healthy normal weight (MHNW), metabolic unhealthy normal weight (MUNW), MHO, and MUO according to expert consensus on the definition of metabolically healthy obesity and screening metabolically healthy obesity in Chinese children [41]. Obesity phenotypes were evaluated on the basis of (1) BMI (normal weight was determined as BMI < 85th percentile and obesity was determined as BMI ≥ 95th percentile for sex- and age-specific group [42]) and (2) metabolic abnormalities ((a) hypertension: SBP and/or DBP ≥ 95th percentile for sex- and age-specific group; (b) elevated fasting glucose: FPG ≥ 5.6 mmol/L; (c) high TG: TG ≥ 1.70 mmol/L; (d) low HDL-C: HDL-C < 1.03 mmol/L). MHNW was defined as normal weight without metabolic abnormalities. MUNW was defined as normal weight with 1–4 metabolic abnormalities. MHO was defined as obesity without metabolic abnormalities. MUO was defined as obesity with 1–4 metabolic abnormalities.

#### 2.3.2. Breastfeeding Duration

Children and adolescents were categorized into three subgroups of 0–5 month(s), 6–11 months, and ≥12 months according to breastfeeding duration (in month). Breastfeeding was not limited to exclusive breastfeeding.

#### 2.3.3. Healthy Lifestyle

Four common obesity risk factors (dietary consumption, physical activity, screen time, and sleep duration) were used to construct a healthy lifestyle score [43,44]. A healthy diet was based on four regularly consumed foods (meat, sugar-sweetened beverage, fruit, and vegetable consumption) linked to childhood obesity [45,46]. In this study, dietary optimum components were defined as daily intake of fruits of ≥3 servings (one serving is about 100 g), vegetables of ≥4 servings (one serving is about 100 g), meat products of 2–3 servings (one serving is about 50 g), and weekly intake of sugar-sweetened beverage of <1 serving (one serving is about 250 mL) according to the dietary guidelines for school-age children in China (2016) [47]. Children and adolescents were considered to have a healthy diet if they consumed at least two of the four common foods in the required servings. Physical activity was categorized by the cutoff of moderate to vigorous physical activity for 1 h per day [48]. Screen time was defined as <2 h of screen time per day and ≥2 h per day. Sleep duration was defined as sufficient sleep and insufficient sleep according to the cutoff of 9 h for children and adolescents [49]. Children and adolescents scored one point for each of the four defined health behaviors. Scores on the lifestyle index ranged from 0–4, and they were further divided into unfavorable lifestyles (zero or one healthy lifestyle factor), intermediate lifestyles (two healthy lifestyle factors), and favorable lifestyles (three or four healthy lifestyle factors) [37].

#### 2.3.4. Confounding Variables

In this study, age, sex, residence, single-child status, delivery model, delivery date, birth weight, family history of diseases, parental education level, parental tobacco and alcohol consumption, maternal age at delivery, and family household income were considered as confounding variables. Children and adolescents were categorized according to their birth weight into low birth weight (LBW, birth weight < 2500 g), normal birth weight (NBW, birth weight: 2500–3999 g), and high birth weight (HBW, birth weight ≥ 4000 g).

### 2.4. Statistics Analysis

Quantitative variables were shown as mean (standard deviation) or median (interquartile range) according to the normality of distribution, and categorical variables were shown as number (percentage). Nonparametric test or Bonferroni multiple comparison method and chi-squared test were used to compare difference of quantitative data and categorical data, respectively, between different breastfeeding duration groups. A multinomial logistic regression model was used to estimate the odds ratio (OR) and 95% confidence interval (95% CI) for the associations between breastfeeding duration and obesity phenotypes, and MHNW was considered as the reference group. The interaction terms of breastfeeding duration and healthy lifestyle category were also tested. When interactions were significant, stratified analyses were performed. The final model was adjusted for age, sex, residence, single-child status, delivery model, delivery time, birth weight, family history of diseases, parental education level, parental tobacco and alcohol consumption, maternal age at delivery, and family household income. All statistical analyses were performed with IBM SPSS Statistics version 26.0 and GraphPad Prism 9. A two-tailed *p*-value <0.05 was considered statistically significant.

## 3. Results

### 3.1. Characteristics of Participants

In the total population, the prevalence of obesity was 11.0%, and the prevalence of MHO and MUO was 4.4% and 6.6%, respectively. Table 1 shows the characteristics of children and adolescents, as well as their parents, by breastfeeding duration groups. A total of 8208 eligible children and adolescents, 2373 in the breastfeeding duration for 0–5 months group, 3184 in the breastfeeding duration for 6–11 months group, and 2651 in the breastfeeding duration for ≥12 months group, were included in the final analysis. Among the children and adolescents with obesity, the prevalence of MHO and MUO was 41.0% and 59.0% respectively.

Compared to the breastfeeding duration for 6–11 months group, children and adolescents who breastfed for ≥12 months had a lower proportion of MHNW (62.0% vs. 69.5%) and a higher proportion of MUNW (38.0% vs. 27.2%); children and adolescents who breastfed for 0–5 months had a higher proportion of MHO (48.9% vs. 37.3%) and a lower proportion of MUO (51.1% vs. 62.7%). A lower proportion of children and adolescents with breastfeeding duration for ≥12 months engaged in a favorable (15.3% vs. 13.5%) and intermediate lifestyle (36.1% vs. 32.6%). Parents in the breastfeeding duration for 0–5 months group had the highest education level (36.2% and 36.4% for junior college or above); however, those in the breastfeeding duration for ≥12 months group had the lowest education level (16.0% and 15.1% for junior college or above). No significant difference was found in monthly household income between different breastfeeding duration groups.

### 3.2. Associations between Breastfeeding Duration and Obesity Phenotypes

**Figure 2:**
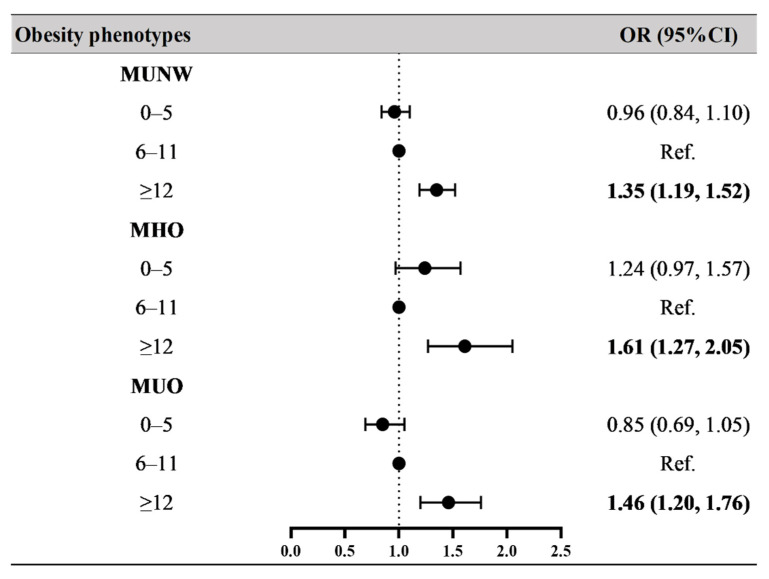
Associations between breastfeeding duration and obesity phenotypes. Obesity phenotypes include metabolic healthy normal weight (MHNW, reference group), metabolic unhealthy normal weight (MUNW), metabolic healthy obesity (MHO), and metabolic unhealthy obesity (MUO). Bold values of OR (95% CI) are statistically significant (*p* < 0.05). Adjusted for age, sex, residence, single-child status, delivery model, delivery date, family history of diseases (obesity, hypertension, diabetes mellitus and cerebrovascular disease), parental education level, parental tobacco and alcohol consumption, maternal age at delivery, and family household income.

Figure 2 and Supplementary Table S1 show the OR values for the associations between breastfeeding duration and obesity phenotypes. Compared to the children and adolescents who were breastfed for 6–11 months, participants who were in the longer breastfeeding duration group (≥12 months) had higher risks of MUNW (OR = 1.35, 95% CI: 1.19–1.52), MHO (OR = 1.61, 95% CI: 1.27–2.05), and MUO (OR = 1.46, 95% CI: 1.20–1.76) after adjusting for age, sex, residence, single-child status, delivery model, delivery date, birth weight, family history of diseases, parental education level, parental tobacco and alcohol consumption, maternal age at delivery, and family household income. In Model 1 (crude model, see Supplementary Table S1), participants who were in the breastfeeding duration for 0–5 months group had a higher risk of MHO (OR = 1.46, 95% CI: 1.16–1.84); however, after adjusting for confounders, these associations turned out to be nonsignificant (Model 3 in Supplementary Table S1).

### 3.3. The Offsetting Effect of Healthy Lifestyle

**Figure 3:**
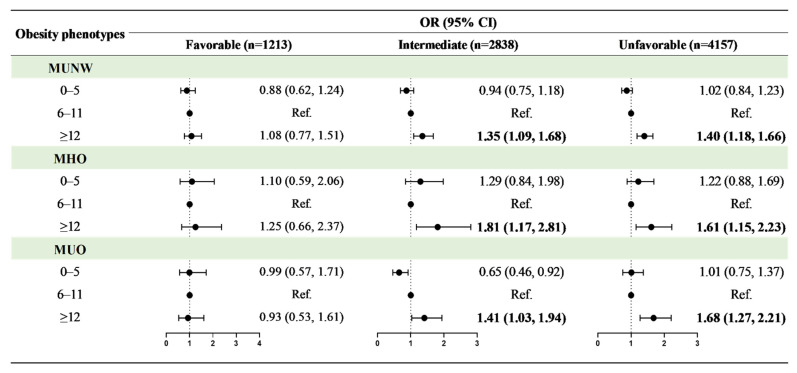
Associations between breastfeeding duration and obesity phenotypes in different healthy lifestyle groups. Obesity phenotypes include metabolic healthy normal weight (MHNW, reference group), metabolic unhealthy normal weight (MUNW), metabolic healthy obesity (MHO), and metabolic unhealthy obesity (MUO). Adjusted for age, sex, residence, single-child status, delivery model, delivery date, birth weight, family history of diseases (obesity, hypertension, diabetes mellitus and cerebrovascular disease), parental education level, parental tobacco and alcohol consumption, maternal age at delivery, and family household income. Bold values of OR (95% CI) are statistically significant (*p* < 0.05).

When breastfeeding duration and healthy lifestyle category were evaluated as interaction variables for obesity phenotypes, it was discovered that healthy lifestyle category and breastfeeding duration for ≥12 months had a significant interaction effect on MUNW, MHO, and MUO (Supplementary Table S2). Figure 3 shows the adjusted OR values for associations between breastfeeding duration and obesity phenotypes stratified by healthy lifestyle category. Although the OR values in certain groups were not statistically significant, there was a typical dose–response relationship between breastfeeding duration for more than 12 months and obesity phenotypes, with increased risks of MUNW (favorable: OR = 1.08, 95% CI: 0.77–1.51; intermediate: OR = 1.35, 95% CI: 1.09–1.68; unfavorable: OR = 1.40, 95% CI: 1.18–1.66), MHO (favorable: OR = 1.25, 95% CI: 0.66–2.37; intermediate: OR = 1.81, 95% CI: 1.17–2.81; unfavorable: OR = 1.61, 95% CI: 1.15–2.23), and MUO (favorable: OR = 0.93, 95% CI: 0.53–1.61; intermediate: OR = 1.41, 95% CI: 1.03–1.94; unfavorable: OR = 1.68, 95% CI: 1.27–2.21) when a favorable lifestyle moved to an unfavorable lifestyle.

## 4. Discussion

This study is the first to report the national prevalence of different obesity phenotypes in China. The prevalence of obesity among Chinese children and adolescents aged 7–18 years was 11.0%. Among the children and adolescents with obesity, the prevalence of MHO and MUO was 41.0% and 59.0%, respectively. On the basis of the different obesity phenotype outcomes, we found that prolonged breastfeeding (≥12 months) was associated with increased risks of MUNW, MHO, and MUO, while a favorable lifestyle may offset the negative effects to some extent.

Consistent with the findings of existing studies [50,51], our results support the conclusion that prolonged breastfeeding is detrimental. Adults of a birth cohort study in Finland who breastfed for 8 months or more reported an 8.8% increase in overweight compared to those who breastfed for a shorter period of time [51]. A prospective cohort study in Sweden found that obesity prevalence was slightly higher for children aged 4 years with breastfeeding for 12 months than those who were breastfed for 9 months [30]. We speculated that the benefits of breastfeeding might begin to wane around the age of 12 months. The physiological mechanism via which prolonged breastfeeding might increase the risk of obesity is chronic exposure to maternal hormones present in breast milk, which could theoretically alter infant lipid metabolism and increase body fat composition later in life [51]. For example, a cohort study in the United Kingdom showed that prolonged breastfeeding was associated with higher cholesterol concentrations in later life [31], while mothers who breastfed for longer than 12 months had increased free thyroxine concentrations [52], which were associated with the regulation of circulating lipoprotein concentrations [53]. In addition, there were U-shaped associations between breastfeeding duration and body fat percentage and blood pressure [31,51]. Infants in poorer families tend to be breastfed for longer [51,54]. For children older than 12 months with a better supply of complementary food in high-income countries, additional breast milk may increase fat intake since the primary nutrient in breast milk after 12 months for infant is fat [55]. These facts may be significant reasons for the higher risk of MHO and MUO in longer periods (≥12 months) of breastfeeding.

A previous study from the same database found that prolonged breastfeeding (≥12 months) was associated with low lipid level and reduced risk of abnormal blood lipids; however, the beneficial effects were mainly reflected in total cholesterol (TC) [56]. Although the OR values of HDL-C and TG were not statistically significant, the HDL-C levels in children and adolescents who were breastfed for more than 12 months were lower than those in the non-breastfeeding group (*p* < 0.01). Only HDL-C and TG overlapped with the metabolic indicators of obesity phenotypes in the present study. Therefore, the results of the two studies are not contradictory, but are presented from different perspectives due to the role of breastfeeding duration in different diseases. Actually, this is the first study in the world to examine the associations between different obesity phenotypes and breastfeeding duration. This study provides a standardized outcome and detailed extensive subgroup analysis, serving as a reference and a more specific association for future investigation.

Breastfeeding for shorter periods of time might be hazardous due to the negative effects of alternate feeding [51]. Nevertheless, in our study, we did not find a role for short-term breastfeeding. The present study discovered that more educated parents established a shorter breastfeeding duration. Highly educated parents may turn to other alternatives such as more intensive care and promoting a healthier lifestyle for their children in later life to prevent obesity. However, the present study did not collect specific information on artificial feeding and food availability. Formula-fed children had higher protein intakes than breastfed children, relative to exclusive breastfeeding [57]. Nevertheless, higher protein intake is thought to increase insulin and insulin-like growth factor-1 (IGF-1) secretion in early childhood, which is associated with early obesity rebound [57]. Breastfed children could automatically control their food intake according to their own requirements, while formula-fed babies were passive. In addition, introducing complementary foods as early as 4 months of age or before increased the risk of overweight in childhood [58]. Thus, the findings may only be applicable to areas with a diverse food market, such as China.

The World Health Organization (WHO) recommends exclusive breastfeeding for 6 months and continued breastfeeding until 2 years or more [59], which is a very ideal goal to reach [60,61], especially in those countries and regions with limited drinking water and food security and diversity [62]. In fact, many Western countries, including 65% of European members and the United States, choose not to fully follow this recommendation [63]. The American Academy of Pediatrics recommends exclusive breastfeeding for 6 months and continued breastfeeding until 1 year or more [64], while guidelines from the European Society of Pediatric Gastroenterology, Hepatology, and Nutrition state that complementary foods could be introduced between the 17th and 26th weeks of life [65]. Moreover, Grummer-Strawn and Rollins emphasized the difficulty of conclusions from breastfeeding research due to the different conclusions of many studies, methodological weaknesses, and the varied mechanisms of breastfeeding affecting health [66]. Despite a systematic review which demonstrated that breastfeeding was linked to a 12% lower incidence of obesity when compared to never breastfeeding [67], this association might no longer be present after controlling for maternal BMI, smoking, and socioeconomic variables, as argued by several well-designed studies [23,27]. Given that varied settings with different social atmospheres, food supplies, and individual characteristics, the recommended breastfeeding duration from WHO might not be suitable for every country, which also need to establish individual policies according to their specific conditions.

Research has shown that, further away from infancy, there is a greater impact of other lifestyle factors (such as diet, exercise) on obesity [21]. When children adhered to a favorable lifestyle in later life, they could have a 50% lower risk of obesity than those who adhered to an unfavorable lifestyle [37]. These findings suggest that the increased risk of childhood obesity induced by prolonged breastfeeding duration might be largely offset by a favorable lifestyle later in life.

Despite these promising results, several limitations remained. Firstly, the associations observed in this study were from a cross-sectional study, leading to weak causal inference. Secondly, due to excessive sample deletion, there may have been some selection bias in the samples of this study. Thirdly, the reliability and validity information of the questionnaire was not provided in this study, and the validity of the questionnaire was not verified. In particular, breastfeeding was not collected according to the scale; thus, there might have been information bias. Fourthly, the information about breastfeeding was retrospectively investigated, and there was a certain recall bias, leading to overestimation or underestimation of effect values. In addition, the breastfeeding group and healthy lifestyle category were artificially divided; thus, misclassification bias existed. Fifthly, the role of formula milk powder and complementary foods in infant feeding was not excluded in this study. Additionally, despite the analysis of many variables that could have led to bias, residual confounding existed. Lastly, this study was not a large-scale national survey, and more regional participation is needed in the future.

## 5. Conclusions

Prolonged breastfeeding (≥12 months) may be associated with increased risks of MUNW, MHO, and MUO, and the benefits of breastfeeding might begin to wane around the age of 12 months among children and adolescents. The increased risks may be largely offset by a favorable lifestyle such as healthy diet, limited screen time, adequate physical activity, and sufficient sleep duration. Breastfeeding duration for no more than 12 months and the establishment of a healthy lifestyle are recommended to prevent different obesity phenotypes for children and adolescents in China. Breastfeeding duration until 2 years or more, as recommended by the WHO, may be amended on the basis of the actual situation in different countries, such as the addition of children’s complementary foods, and policies in the light of evidence-based studies should be formulated.

## Figures and Tables

**Table 1:** Demographic characteristics of eligible children and adolescents and their parents, stratified by breastfeeding duration (*n* = 8208).

	Breastfeeding Duration (Months)				*p*-Value
	Total	0–5 (*n* = 2373)	6–11 (*n* = 3184)	≥12 (*n* = 2651)	
Characteristics of children and adolescents					
Age *	11.2 (4.0)	11.1 (6.0)	11.2 (6.0)	11.3 (4.0)	0.016
Sex					0.013
Boys	3914 (47.7)	1180 (49.7) ^#^	1458 (45.8)	1276 (48.1)	
Girls	4294 (52.3)	1193 (50.3) ^#^	1726 (54.2)	1375 (51.9)	
Residence					<0.001
Urban	4623 (56.3)	1533 (64.6) ^#^	1844 (57.9)	1246 (47.0) ^#^	
Rural	3585 (43.7)	840 (35.4) ^#^	1340 (42.1)	1405 (53.0) ^#^	
Obesity phenotypes					
Normal weight					<0.001
MHNW	4776 (68.1)	1472 (72.8) ^#^	1927 (69.5)	1377 (62.0) ^#^	
MUNW	2242 (31.9)	551 (27.2) ^#^	847 (30.5)	844 (38.0) ^#^	
Obesity					0.002
MHO	488 (41.0)	171 (48.9) ^#^	153 (37.3)	164 (38.1)	
MUO	702 (59.0)	179 (51.1) ^#^	257 (62.7)	266 (61.9)	
Healthy lifestyle category					0.001
Favorable	1213 (14.8)	367 (15.5)	487 (15.3)	359 (13.5) ^#^	
Intermediate	2838 (34.6)	825 (34.8)	1150 (36.1)	863 (32.6) ^#^	
Unfavorable	4157 (50.6)	1181 (49.8)	1547 (48.6)	1429 (53.9)	
Single-child status					<0.001
Single children	5493 (66.9)	1733 (73.0) ^#^	2188 (68.7)	1572 (59.3) ^#^	
Non-single children	2715 (33.1)	640 (27.0) ^#^	996 (31.3)	1079 (40.7) ^#^	
Delivery time					<0.001
Normal	3802 (46.3)	1083 (45.6) ^#^	1340 (42.1)	1379 (52.0) ^#^	
Premature delivery	2435 (29.7)	803 (33.8)	993 (31.2)	639 (24.1) ^#^	
Delayed delivery	1971 (24.0)	487 (20.5) ^#^	851 (26.7)	633 (23.9) ^#^	
Delivery model					<0.001
Caesarean	3428 (41.8)	1192 (50.2) ^#^	1288 (40.5)	948 (35.8) ^#^	
Eutocia	4780 (58.2)	1181 (49.8) ^#^	1896 (59.5)	1703 (64.2) ^#^	
Birth weight					<0.001
NBW	6881 (83.8)	1954 (82.3)	2665 (83.7)	2262 (85.3)	
LBW	569 (6.9)	213 (9.0)	233 (7.3)	123 (4.6) ^#^	
HBW	758 (9.2)	206 (8.7)	286 (9.0)	266 (10.0)	
Family history of diseases ^$^					0.022
Yes	1254 (15.3)	403 (17.0) ^#^	459 (14.4)	392 (14.8)	
No	6954 (84.7)	1970 (83.0) ^#^	2725 (85.6)	2259 (85.2)	
Parental characteristics					
Paternal education level					<0.001
Primary or below	581 (7.1)	133 (5.6)	169 (5.3)	279 (10.5) ^#^	
Secondary or equivalent	5338 (65.0)	1381 (58.2) ^#^	2010 (63.1)	1947 (73.4) ^#^	
Junior college or above	2289 (27.9)	859 (36.2) ^#^	1005 (31.6)	425 (16.0) ^#^	
Maternal education level					<0.001
Primary or below	769 (9.4)	159 (6.7) ^#^	273 (8.6)	337 (12.7) ^#^	
Secondary or equivalent	5221 (63.6)	1351 (56.9) ^#^	1956 (61.4)	1914 (72.2) ^#^	
Junior college or above	2218 (27.0)	863 (36.4) ^#^	955 (30.0)	400 (15.1) ^#^	
Paternal tobacco consumption					<0.001
Yes	4327 (52.7)	1220 (51.4)	1612 (50.6)	1495 (56.4) ^#^	
No	3881 (47.3)	1153 (48.6)	1572 (49.4)	1156 (43.6) ^#^	
Maternal tobacco consumption					0.001
Yes	97 (1.2)	38 (1.6) ^#^	20 (0.6)	39 (1.5) ^#^	
No	8111 (98.8)	2335 (98.4) ^#^	3164 (99.4)	2612 (98.5) ^#^	
Paternal alcohol consumption					0.120
Yes	2560 (31.2)	732 (30.8)	962 (30.2)	866 (32.7)	
No	5648 (68.8)	1641 (69.2)	2222 (69.8)	1785 (67.3)	
Maternal alcohol consumption					0.453
Yes	152 (1.9)	48 (2.0)	62 (1.9)	42 (1.6)	
No	8056 (98.1)	2325 (98.0)	3122 (98.1)	2609 (98.4)	
Maternal age at delivery *	26.4 (4.8)	27.0 (4.8) ^#^	26.1 (4.7)	26.1 (4.8)	<0.001
Monthly household income					0.835
<5000 CNY	6803 (82.9)	1958 (82.5)	2641 (82.9)	2204 (83.1)	
≥5000 CNY	1405 (17.1)	415 (17.5)	543 (17.1)	447 (16.9)	

Note: Categorical variables are expressed by frequency value (percentage, %). * Quantitative variables are shown as median (interquartile range) because the data did not follow a normality of distribution. ^$^ Family history of diseases includes obesity, hypertension, diabetes mellitus, and cerebrovascular disease. ^#^ Significant difference compared with the breastfeeding duration for 6–11 months group. MHNW, metabolic healthy normal weight; MUNW, metabolic unhealthy normal weight; MHO, metabolic healthy obesity; MUO, metabolic unhealthy obesity; NBW, normal birth weight; LBW, low birth weight; LBW, high birth weight.

## Data Availability

The data supporting the conclusions of this article can be made available from the corresponding author upon request.

## Supplementary Materials

The following supporting information can be downloaded at https://www.mdpi.com/article/10.3390/nu14101999/s1: Table S1. Associations between breastfeeding duration and obesity phenotypes; Table S2. Risk of obesity phenotypes according to healthy lifestyle category and breastfeeding duration.
