# Supplementary material for: Associations between Breastfeeding Duration and Obesity Phenotypes and the Offsetting Effect of a Healthy Lifestyle
Source: Nutrients. 2022 May 10;14(10):1999. doi: 10.3390/nu14101999 (PMC9143350; doi:10.3390/nu14101999)
Supplement: Supplementary file 1 [file nutrients-14-01999-s001.zip › nutrients-1687627-supplementary.pdf]

Table S1. Associations between breastfeeding duration and obesity phenotypes.

Obesity phenotypes [a]	Breastfeeding duration (months)	No. of cases	OR (95% CI)		
			Model 1 [b]	Model 2 [c]	Model 3 [d]
MUNW	0–5	551	1.00 (0.89, 1.12)	0.94 (0.83, 1.07)	0.96 (0.84, 1.10)
	6–11	847	Ref.	Ref.	Ref.
	≥12	844	**1.39 (1.24, 1.57)**	**1.43 (1.27, 1.62)**	**1.35 (1.19, 1.52)**
MHO	0–5	171	**1.46 (1.16, 1.84)**	**1.38 (1.09, 1.74)**	1.24 (0.97, 1.57)
	6–11	153	Ref.	Ref.	Ref.
	≥12	164	**1.50 (1.19, 1.89)**	**1.55 (1.23, 1.96)**	**1.61 (1.27, 2.05)**
MUO	0–5	179	0.91 (0.74, 1.12)	0.89 (0.73, 1.10)	0.85 (0.69, 1.05)
	6–11	257	Ref.	Ref.	Ref.
	≥12	266	**1.45 (1.20, 1.74)**	**1.42 (1.18, 1.71)**	**1.46 (1.20, 1.76)**

[a] Obesity phenotype I includes Metabolic Healthy Normal Weight (MHNW, Reference group), Metabolic Unhealthy Normal Weight (MUNW), Metabolic Healthy Obesity (MHO) and Metabolic Unhealthy Obesity (MUO).

[b] Model 1: crude model.

[c] Model 2 adjusted for age, sex and residence of children and adolescents.

[d] Model 3 adjusted for variables in Model 2 plus single-child status, delivery model, delivery date, birthweight, family history of diseases (obesity, hypertension, diabetes mellitus and cerebrovascular disease), parental education level, parental tobacco and alcohol consumption, maternal age at delivery, family household income. Bold OR (95% CI) indicated statistically significant ($P < 0.05$).

Table S2. Risk of obesity phenotypes according to healthy lifestyle category and breastfeeding duration.

Breastfeeding duration (months)	Healthy lifestyle category	MUNW		MHO		MUO	
		Adjusted OR (95% CI)	*P* value	Adjusted OR (95% CI)	*P* value	Adjusted OR (95% CI)	*P* value
0–5	Favorable	0.84 (0.60, 1.16)	0.288	1.07 (0.58, 1.95)	0.831	0.87 (0.51, 1.47)	0.598
	Intermediate	0.79 (0.60, 1.04)	0.091	1.07 (0.64, 1.78)	0.795	0.83 (0.54, 1.28)	0.402
	Unfavorable	0.97 (0.75, 1.25)	0.813	1.38 (0.86, 2.21)	0.188	1.08 (0.72, 1.62)	0.715
6–11	Favorable	Ref.		Ref.		Ref.	
	Intermediate	0.92 (0.71, 1.19)	0.522	0.8 (0.48, 1.33)	0.388	1.24 (0.83, 1.84)	0.289
	Unfavorable	1.10 (0.87, 1.40)	0.430	1.1 (0.68, 1.78)	0.690	1.04 (0.70, 1.54)	0.853
≥12	Favorable	1.13 (0.82, 1.56)	0.457	1.3 (0.70, 2.40)	0.406	0.98 (0.57, 1.68)	0.946
	Intermediate	1.29 (0.99, 1.67)	0.063	1.49 (0.89, 2.49)	0.132	**1.76 (1.17, 2.65)**	0.007
	Unfavorable	**1.51 (1.18, 1.93)**	0.001	**1.71 (1.06, 2.75)**	0.028	**1.70 (1.16, 2.52)**	0.007

Note: Obesity phenotype I includes Metabolic Healthy Normal Weight (MHNW, Reference group), Metabolic Unhealthy Normal Weight (MUNW), Metabolic Healthy Obesity (MHO) and Metabolic Unhealthy Obesity (MUO). Adjusted for age, sex, residence, single-child status, delivery model, delivery date, birthweight family history of diseases (obesity, hypertension, diabetes mellitus and cerebrovascular disease), parental education level, parental tobacco and alcohol consumption, maternal age at delivery, family household income.
